# The Toxic Effects of Xenobiotics on the Health of Humans and Animals

**DOI:** 10.1155/2017/4627872

**Published:** 2017-03-29

**Authors:** Yanzhu Zhu, Alex Boye, Mathilde Body-Malapel, Jorge Herkovits

**Affiliations:** ^1^Key Laboratory of Special Animal Epidemic Disease of Ministry of Agriculture, Institute of Special Animal and Plant Sciences, Chinese Academy of Agricultural Sciences, Changchun 130112, China; ^2^University of Cape Coast, Cape Coast, Ghana; ^3^Université Lille, Inserm, CHU Lille, U995-LIRIC-Lille Inflammation Research International Center, 59000 Lille, France; ^4^Instituto de Ciencias Ambientales y Salud, Buenos Aires, Argentina

With the development of the society, the xenobiotics have brought high potential risk to human and animal. The study focused on the adverse health effect of xenobiotics which plays an important role in addressing public health challenge. The special issue has focused on the toxicity of xenobiotics. We call for manuscripts describing recent findings and future perspectives in the toxic field of xenobiotics on the human and animal health. Until the deadline of the special issue, 20 manuscripts have been received worldwide. After the review process, 7 manuscripts have been accepted by this special issue. The accepted research manuscripts have focused on the insecticides (Organochlorine Pesticides, Furia ®180 SC (Zeta-cypermethrin) and Bulldock 125®SC (*β*-Cyfluthrin) pyrethroid insecticides), sterilant (zinc gluconate-based chemical sterilant), toxic component of plant (usnic acid from* Cladonia substellata* Vainio and swainsonine from locoweed), and ethyl maltol and iron complexes. The accepted review manuscripts have described the enzymes and proteins which contributed to IDILI and the various assays and current trends in developing comprehensive models. They represent the important research finding and will contribute to the development of toxicology.

*Insecticides* bring high risk to human and animal through food, water, and air. The World Health Organization have estimated 3 million cases of severe acute poisoning (including suicides) and reported cases of intoxication responsible for approximately 2.2 million deaths. The number of incidents of exposure to pyrethroid insecticides has increased. Organochlorinated compounds (OCPs) are one of the important pesticides and have been used globally for many years. OCPs are not efficiently eliminated from the human body and may accrue in tissues because of enterohepatic reabsorption and affinity to adipose tissue. There has been limited work on interventions to facilitate elimination of OCPs. Evidence confirmed that enhanced mobilization and excretion via induced perspiration reduced the body burden of hexachlorobenzene (HCB) and polychlorinated biphenyl congeners. Removal of OCPs has also been facilitated by specific interventions which interrupts the enterohepatic circulation. To confirm that, sweat analysis may be useful in detecting some accrued OCPs, not found in regular serum testing. This method supplements the detection method of the OCPs. But perspiration is hard to collect. It maybe restricts its extensive use. Pyrethroid insecticides are another one of the important pesticides. Pyrethroid insecticides represent more than 30% of the worldwide insecticide market. Furia 180 SC is an isomer of cypermethrin insecticide. Bulldock 125 SC is an isomer of cyfluthrin. However, there is little information concerning the toxic effects of zeta-cypermethrin. But the research on the toxic effect of the* Furia 180 SC (Zeta-cypermethrin)* and Bulldock 125 SC (*β*-cyfluthrin) is still necessary in the future.

Sclerosing agents as zinc gluconate-based chemical sterilants (Infertile®) are used for chemical castration, but there is not enough evidence of its safety profiles for the receivers. Infertile at 60 mM presented risk for animal health. It suggests that the optimal dose must be less than 6 mM. The future studies must be continued in order to clarify the safety of Infertile on cells and tissues, in order to better understand the pathophysiological mechanisms of this drug.

The toxic component of plant often attracts the attention from food safety. Usnic acid is a compound of natural origin resulting from lichen secondary metabolism. It is considered one of the most important biologically active metabolites with important pharmacological properties: antitumor, antibiotic, antiviral, antioxidant, tuberculostatic, anti-inflammatory, and molluscicide. But limited studies showed toxicological potential of usnic acid. The teratogenic effect of usnic acid is in the period of organogenesis. It reveals the importance of evaluating the toxic effects of natural substances for elucidating the care in their indication as drug, particularly during pregnancy. Swainsonine is the primary toxin in locoweeds which is toxic plants. Its toxicity often occurs during pregnancy in livestock, and it may have different effects on embryonic development depending on the conceptus phase and maternal conditions during acute intoxication. Limited data showed the potential reproductive and developmental toxicity caused by swainsonine. The swainsonine caused reproductive and developmental toxicity in both parents and offspring mice. It suggests that severe reproductive toxicity and developmental toxicity are associated with swainsonine poisoning.

Ethyl maltol and iron complexes are products of ethyl maltol and the iron found in the cooking pots used to prepare the Chinese dish, hot-pot. Limited data showed their safety. The ethyl maltol and iron complexes were toxic, and the targeted endocrine organs were the liver and kidneys. It suggests that alternative strategies for preparing the hot-pot, including the use of non-Fe-based cookware, need to be developed and encouraged to avoid the formation of the potentially toxic complexes. This experiment focused on the toxic of ethyl maltol and iron complexes, which is related to the Chinese dish, hot-pot. It is significant and it will attract more attention on these complexes.

Idiosyncratic drug-induced liver injury (IDILI) is a significant source of drug recall and acute liver failure (ALF) in the United States. While current drug development processes emphasize general toxicity and drug metabolizing enzyme- (DME-) mediated toxicity, it has been challenging to develop comprehensive models for assessing complete idiosyncratic potential. The enzymes and proteins contributed to IDILI, including ones that affect phase I and phase II metabolism, antioxidant enzymes, drug transporters, inflammation, and human leukocyte antigen (HLA) which are important. It is imperative to develop various assays to detect individual reactions focusing on each of the mechanisms. The current trends in developing comprehensive models focused on examining the mechanisms. This review about IDILI is comprehensive and deeply showed the literature of potential mechanisms and predictive assays in idiosyncratic drug-induced liver injury. This review is significant and presents high value.

